# Effects of muscle dysmorphia, social comparisons and body schema priming on desire for social interaction: an experimental approach

**DOI:** 10.1186/s40359-017-0189-9

**Published:** 2017-06-15

**Authors:** Catharina Schneider, Maria Agthe, Takuya Yanagida, Martin Voracek, Kristina Hennig-Fast

**Affiliations:** 1 Department of Applied Psychology: Health, Development, Enhancement, and Intervention, Faculty of Psychology, University of Vienna, Vienna, Austria; 2 Department of Psychology, Ludwig-Maximilians University Munich, Munich, Germany; 3 School of Medical Engineering and Applied Social Sciences, University of Applied Sciences Upper Austria, Wels, Austria; 4 Department of Basic Psychological Research and Research Methods, Faculty of Psychology, University of Vienna, Vienna, Austria; 5 Department of Psychiatry and Psychotherapy, Evangelisches Krankenhaus Bielefeld, Bielefeld, Germany

**Keywords:** Muscle dysmorphia, Social interaction, Social comparisons, Body schema, Experimental design

## Abstract

**Background:**

Muscle dysmorphia (MD) is a relatively young diagnosis referring to the desire for a high degree in lean muscle mass, while simultaneously believing that one is insufficiently muscular, mostly found in men. It goes along with a risk for social withdrawal to maintain rigid exercise and dietary regimen. The aim of the current study was thus, to explore differences in men with and without a risk for muscle dysmorphia regarding their desire for social interaction. Furthermore, we investigated potential effects of individual social comparison tendencies (the tendency to compare oneself with persons who are perceived to be superior or inferior to oneself on a certain dimension) and of one’s own body schema on the desire for social interaction.

**Methods:**

One hundred physically active, college aged Austrian men were recruited via social media and flyers at fitness centers and the sports department of the University of Vienna. Participants were randomly assigned to a priming condition evoking their own body schema or a control condition and had to state their desire for social interaction with male or female stimulus persons of high or average attractiveness. We conducted a 2 (group of participant; men with vs. without a risk for MD) × 2 (priming condition; priming vs. non-priming) × 2 (attractiveness of stimulus person; highly attractive vs. less attractive) experimental design with different social comparison tendencies as covariates.

**Results:**

Men with a risk for muscle dysmorphia showed lesser desire for social interaction than men without this risk, which can be seen as a risk factor for psychopathological outcomes. Generally, men with and without a risk for muscle dysmorphia did not differ with regard to their preferences for attractive stimulus persons as subjects for social interaction. We confirmed the notion that a tendency for downward social comparisons goes along with a diminished desire for social interaction.

**Conclusions:**

This study showed that men with a risk for muscle dysmorphia appeared to be at higher risk for social withdrawal and that this is associated with social comparison tendencies. Future investigations on clinical populations are needed, for this population is highly prone to social isolation and negative outcomes related to it.

## Background

### Muscle dysmorphia

Muscle dysmorphia (MD) is a rare phenomenon which receives growing interest in the scientific community. It refers to the pathological desire to increase lean muscle mass and the simultaneous believe of being insufficiently muscular [1]. Although positioned within body dysmorphic disorders in the DSM-5 [2], similarities with eating disorders, especially anorexia nervosa (AN), have been postulated [3–7]. One important similarity might be the tendency for social isolation, as was postulated for women with AN [2, 8], as well as for men with MD [9, 10]. Like one diagnostic feature of body dysmorphic disorders [2], MD may cause impairment in social and occupational functioning [11]. It has been described that individuals with MD spent long hours exercising, invest excessive attention to their diet, and give up other social, occupational or recreational activities, such as eating at restaurants, because the caloric information on the food is lacking [10]. Individuals with MD reported to decline social invitations or refuse to be seen at the beach out of fear of looking to small [12]. Those situations of bodily exposure are often marked by distress or intense anxiety. Some are even housebound for several days, because they feel so bad about their body shape that they do not want to be seen by others [10]. Pope and colleagues [11] also reported that persons with MD can have problems regarding their intimate relationships, resulting from embarrassment about their bodies or fear of rejection of their partners [13]. Some even forgo intimate relationships or occupational opportunities, because another person or job could compromise their exercise and diet regimen [11].

Additionally, it has been found that for men with MD social body comparisons with others are very important. These comparisons seem to function as mediating factors between sociocultural influences and muscularity-oriented body dissatisfaction in men, potentially leading to risky body change behaviors [14].

### Social comparisons and the desire for social interaction

Social comparison theory, as originally suggested by Festinger [15], states that in order to form assessments of themselves, individuals compare themselves to others on characteristics important to them. Men with body image concerns, therefore, may compare themselves with other men in order to learn more about the ideal shape of their bodies [14].

In addition to Festingers’ original postulation, it was found that people tend to engage in comparison strategies related to underlying motives of self-enhancement and self-protection [16]. When self-evaluation is threatened, people lean towards comparisons with persons who are worse off than themselves (e.g., engage in downward comparisons), thereby serving self-protection [17].

In general, people who have an inclination to engage in downward comparisons tend to be more susceptible to self-threat and they are more likely to experience averse contrast effects. Therefore, the seeking of downward comparisons of those who feel particularly threatened by others aims at reducing self-evaluative threats [18]. Yet, not all persons avoid upward social comparisons. Some people generally compare upwards, as they tend to be interested in self-improvement. Consequently, exposure to physically attractive persons should be less threatening [19]. In fact, people’s social comparison orientation (i.e., tendencies for upward vs. downward comparison) has been found to moderate their reactions to others who may pose a self-threat to oneself in such comparison (e.g., regarding physical attractiveness; [20]). Yet, when self-improvement fails, self-evaluative threat may be high [21].

Referred to body image, this might be of special importance in populations with body image problems and (related) low self-esteem, because unsuccessful upward social comparisons could lead to even more body dissatisfaction and even lower self-esteem, thereby possibly leading to a downward spiral. In line with these considerations, studies found that women with eating disorders engaging in upward appearance-based comparisons were at higher risk for body dissatisfaction and disordered eating [21, 22]. For men, a strong tendency to compare oneself to others exacerbated the relationship of body dissatisfaction and drive for muscularity [23]. Moreover, social body comparisons were related to men’s body dissatisfaction and body change behavior [14], as well as to their drive for muscularity [24].

Social comparison processes, having an influence on self-evaluation outcomes, may as well have an influence on the desire for social interaction with different kinds of people. For instance, several studies in social and organizational contexts showed that people’s evaluations of other persons, as well as their desire to socially interact with others, depends on stimulus persons’ attractiveness [20, 25].

### Body schema

MD goes along with a distorted body image (affected individuals perceive their bodies to be insufficiently muscular). Body image is a multidimensional construct, defined by the perception of and attitudes (cognitive and emotional) about one’s body [26]. Therein, self-schemas can be conceptualized as a cognitive aspect of body image. Self-schemas are understood as cognitive generalizations of ones’ self. They are conveyed from past experiences and organize self-related information processing [27]. A person, for whom appearance is important, will develop more complex networks of knowledge concerning appearance and will be more prone to information-processing biases related to this self-schema (affecting attention, memory and judgment related to body image; [28]).

To evoke body schemas in a person, different approaches have been used. Generally, body schemas have been provoked through questions regarding body image, commercials showing models with “ideal” bodies, or ads for beauty products [28]. Although models in magazines activate body schemas, it could also be assumed that they initiate social comparison processes, since for the investigation of comparisons similar procedures have been used [21, 22]. Another approach postulated that body exposure by mirror confrontation can provoke (negative) body schema [29]. To investigate the effect of body schema, the confrontation with the own body (e.g. via an individual, but standardized photograph, along with a figure-rating scale) seems a plausible way to allow an investigation of the effects of body schema and social comparison separately.

### Aim

In the current study, we investigated whether men with a high versus low risk for MD differ in their desire for social interaction with others. We hypothesized firstly that, men who display a risk for MD report less desire for social interaction than men without a risk for MD. Secondly, in line with Försterling et al. [25], we predict that, regardless of their respective risk for MD, men would generally prefer to interact with attractive rather than less attractive persons. According to the attractiveness halo effect [30, 31], attractive persons are preferred as interaction partners. Thirdly, we investigated whether (a) the tendency for upward- or (b) downward social comparisons as well as (c) the importance of a positive outcome of social comparisons would moderate this desire for social interaction. According to Agthe and colleagues [20], we expected the tendency for upward social comparisons to be related to more desire for social interaction and downward social comparisons to be related to less desire for social interaction with others. The desire to protect one’s self-esteem (i.e., in this case, the importance of a positive outcome of social comparisons) is likely to be related to a lesser desire for interaction with others (particularly men who are attractive, as attractive same-sex persons are more likely to be perceived as self-threat and potential rivals).

Furthermore, our fourth hypothesis postulates an effect of self-reflection and salience regarding one’s own body on the desire for social interaction. Given that it is the feeling of being too small or insufficiently muscular that often causes social withdrawal [10–12], it seems plausible that the activation and corresponding salience of one’s own body schema could lead to less desire for social interaction. Therefore, using a priming condition, we investigated the immediate effect of an activated body schema on the desire for social interaction in men with and without a risk for MD.

## Method

### Participants

One hundred and four men were invited to participate in the study. Four persons had to be excluded due to insufficient command of the German language or as a result from technical problems. Accordingly, the final sample consisted of 100 individuals.

The average age of the participants was 24.2 years (*SD* = 3.8), their height was 1.80 m (*SD* = 7.21) and weight was 82 kg (*SD* = 10.6). The sample contained predominantly university students (71), 23 participants were working, two were unemployed, three were in high-school, and one reported to be a professional athlete. All men stated to identify as heterosexual. Fifty men reported to be single and fifty to be in a relationship. As shown in Table 1, 52 men were assigned to the priming condition and 48 received no priming. Screened for MD, 23 individuals were detected to be at risk for MD versus 77 men without a risk for MD.

**Table 1:** Groups: Risk for MD and priming condition

	priming	no priming	total
No risk for MD	41	36	77
risk for MD	11	12	23
Total	52	48	100

*MD* muscle dysmorphia

### Materials

Data were collected as part of a bigger study on male body image.

#### Sociodemographic measures

Once informed consent was gained, sociodemographic data (e.g., nationality, age, sexual orientation, educational qualification, relationship status) were collected.

#### Screening for muscle dysmorphia

In addition, we screened respondents for MD by using four screening questions, representing the main symptoms of MD according to Pope and colleagues [11]. The main symptoms are preoccupation with the idea of being insufficiently lean and muscular, giving up social, occupational, or recreational activities to maintain workout and diet schedule, and clinically significant distress caused by the preoccupation of being insufficiently muscular. Items 6 and 11 of the Muscle Dysmorphia Inventory (MDI) [32] as well as items 17 and 18 from the Muscle Dysmorphic Disorder Inventory (MDDI) [33] were translated into German and rated on a six-point scale from 1 (*never*) to 6 (*always*). Items were (1) *I am concerned with losing muscle mass*, (2) *I am preoccupied that I look to small*, (3) *I pass up social activities (*e.g. *watching football games, eating dinner, going to see a movie,* etc.*) with friend because of my workout schedule*, (4) *I feel depressed when I miss one or more workout days*. Cronbach’s α for the screening scale was .75. To distinguish men with and without a risk for MD, a cut-off value was used. Since it was assumed that men with a risk for MD would report most of these symptoms no less than *often* (value of 4), contrary to men without a risk for MD who probably report to experience these symptoms *never* (1), *rarely* (2), or *sometimes* (3)) the cut-off value of 16 was established. Thus, individuals had to report to experience at least three out of four symptoms of MD no less than *often* and in case they report one lesser than *often*, at least one other symptom must be rated more than *often* to reach the cut-off value.

#### Social comparisons

According to Agthe and colleagues [20], we assessed tendencies for upward- or downward social comparisons with three self-developed questions, asking whether persons tend to compare themselves with others whom they perceive to be (1) superior or (2) inferior to themselves. Additionally, we asked (3) how important it is for them to get a positive outcome in these comparisons. The three questions had to be answered on a five-point rating scale, ranging from 1 (*not at all*) to 5 (*totally*).

#### Desire for social interaction

As part of the experimental design, participants had to rate their desire for social interaction with a male or female, highly attractive or less attractive stimulus person. Items were, for example, *If I’d had the chance, I would like to meet him/her.* All items were rated on a seven-point rating scale, ranging from 1 (*not at all*) to 7 (*very much*).

### Procedure

Participants were recruited via various social media platforms (e.g., for sport students, weight trainers, and recreational athletes) and folders displayed at different fitness studios, sport clubs and the University Sport Department. Due to the experimental design, only heterosexual men, fluent in the German language, who were exercising (participating in their sport) at least three times a week, were invited. The study took place at the Faculty of Psychology at the University of Vienna. After applying via e-mail, potential participants were contacted and screened for exclusion criteria (e.g., homosexuality, insufficient command of the German language, participation in sport less than three times a week) and an appointment was made. After arriving at the Faculty, they gave written informed consent. For the priming condition, they were photographed in a standardized manner, dressed in a black sleeveless shirt and running pants which were provided. The participants were pseudo-randomly assigned to either the priming or the non-priming condition (in order to receive similar group sizes, every second applicant for the study was assigned to the priming condition). Afterwards, they read the cover story. To disguise the intention of the study, participants were told that the experiment was designed to explore whether different sports and one’s body image influence the evaluation of and interest in various professions. The questionnaires, priming, and experimental design were presented on a computer screen and had to be filled out online. Participants were given 30 € as incentive to participate in the study and were thoroughly debriefed afterwards.

#### Experimental design

To investigate the effects of risk for MD, tendencies for social comparisons and body schema on the desire for social interaction, we used an experimental design, build on prior research [20]. The desire for social interaction was the dependent variable, while risk for MD and body schema were introduced as independent variables. Furthermore, we used the attractiveness of the stimulus person as independent variable to investigate potential effects of other persons’ attributes on the participants’ desire for social interaction. Different tendencies for social comparison were integrated as potential moderator variables. Thus, the experiment was based on a 2 (group of participant; men with vs. without a risk for MD) × 2 (priming condition; priming vs. non-priming) × 2 (attractiveness of stimulus person; highly attractive vs. less attractive) between-subject design. To manipulate the stimulus person’s attractiveness, pretested pictures of an highly attractive and a less attractive male or female stimulus person were used [34]. In an on-screen questionnaire, participants were asked to answer questions regarding a stimulus person whose picture (male vs. female; attractive vs. less attractive) was presented in the questionnaire and who was described in the text. This information was kept identical in all conditions with the exception of the first name of the stimulus person, which was different for male vs. female stimulus persons. The female character was introduced as Daniela G. and the male character was called Daniel G. To disguise the intentions of the study, participants were informed that the study aimed at investigating the influence of different sports and one’s body image on the evaluation of and interest in various professions. These professions were, for example, corporative, creative, manual, and social professions. The stimulus person was introduced as working in an advertising agency (creative profession) for two years. S/he had gathered experience before as an intern in the same company, and after his/her Master’s degree, s/he was hired as creative director. S/he likes his/her job because of the possibility to work with different people and the chance to find creative solutions for everyday challenges. Additionally, the participants were given information about the stimulus person’s alleged hobbies and interests.

As a manipulation check, participants rated on a seven-point rating scale how attractive they perceived the stimulus person to be. Then, participants answered various questions regarding the stimulus person (e.g., attributing their success to internal or external factors, whether they like them or would like to have the same job as them). Most importantly, they indicated their desire for social interaction with the respective stimulus person.

#### Priming

**Fig. 1:**
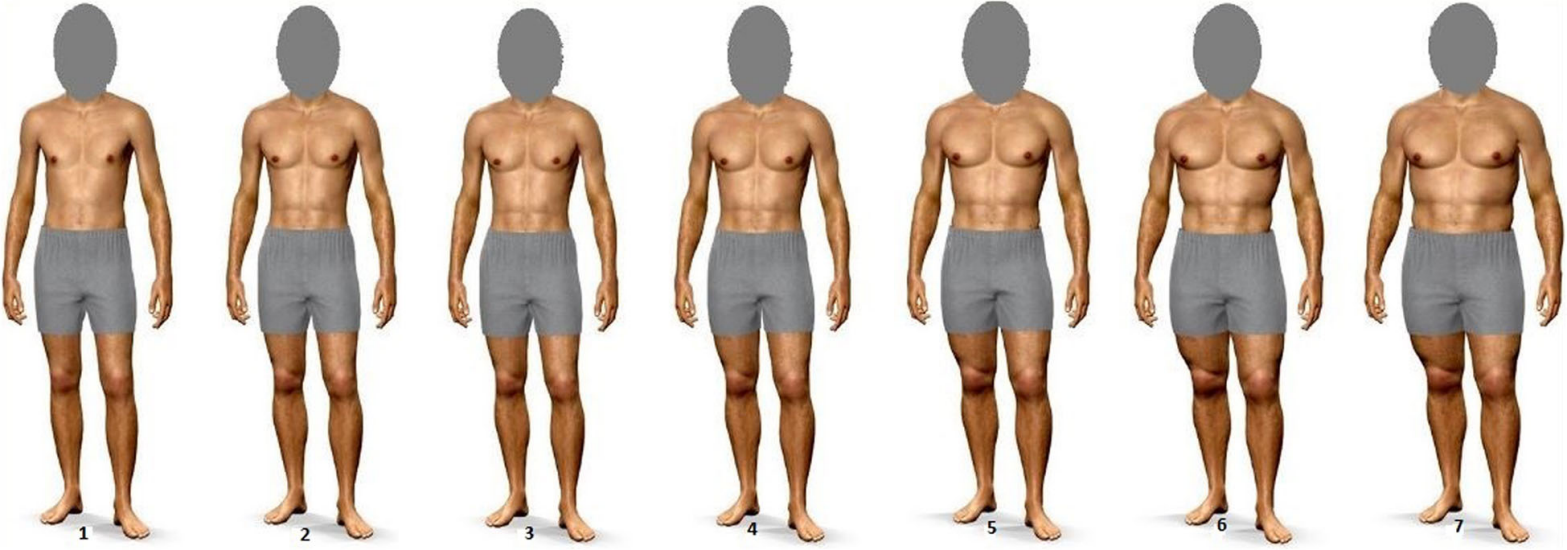
Figure rating scale. Note The images were created using modelmydiet.com

To assess a potential effect of the participants’ own body schema on the experimental design, we used a priming task just before the experimental design (i.e., before participants were presented the stimulus persons and indicated their reactions toward them). The priming consisted of the photographic image of the participant from head downward, which was taken directly before the testing and was presented on screen for ten seconds, without the possibility to skip forward to the next page to control for potential confounding effects. Additionally, we used a picture rating scale, on which the participant had to rate his actual and his desired body shape. Analogous to Frederick and Haselton [35], images for the rating scale where generated via modelmydiet.com, a program allowing to manipulate the physical features of a virtual model (see Fig. 1). Except from muscularity and weight, all other features were kept constant. The scale consists of seven images from skinny/non-muscular to large/muscular.

Participants were pseudo-randomly assigned to the priming or non-priming condition (every second participant was assigned to the priming condition). The latter group received the photograph and picture rating scale after the experiment. Thus, picture and rating scale have no effect on the experimental design, while body schema related data were still available.

All work was approved by the Ethics Committee of the University of Vienna. All participants gave written informed consent prior to study begin.

## Results

### Manipulation check

The participants rated the attractive stimulus persons to be substantially more attractive (male: *M* = 5.24, *SD* = 0.67; female: *M* = 5.94, *SD* = 0.72) than the less attractive ones (male: *M* = 2.74, *SD* = 1.04; female: *M* = 2.71, *SD* = 1.22), *F*(1, 96) = 80.28, *p* < .001, η_p_^2^ = .72, showing that the attractiveness manipulation was effective.

### Group differences

As shown in Table 2, a 2 (risk for MD: high vs. low) × 2 (priming vs. no priming) × 2 (stimulus person attractiveness: high vs. low) analysis of variance on desire for social interaction revealed a significant main effect of risk for MD, *F*(1, 99) = 5.65, *p* < .05, η_p_^2^ = .06 and attractiveness, *F*(1, 99) = 14.65, *p* < .001, η_p_^2^ = .14.

**Table 2:** Means and standard deviation of participants’ desire for social interaction for main effects

Variable		desire for social interaction with SP				
		*M*	*SD*	*F*	η_p_^2^	*d*
MD	risk for MD	12.35	5.75	5.65*	.06	−0,516
	no risk for MD	15.32	5.77			
Priming	priming	14.52	5.66	0.12	.00	−0.042
	no priming	14.77	6.16			
SP attractiveness	attractive	16.88	5.36	14.65**	.14	0.821
	less attractive	12.40	5.55			

*MD* muscle dysmorphia, *SP* stimulus person

* *p* < .05 ** *p* < .01

There was no significant main effect of priming *F*(1, 99) = 0.12, *p* = .73, η_p_^2^ = .00, but one significant interaction with stimulus persons attractiveness, *F*(1, 99) = 9.4, *p* < .01, η_p_^2^ = .09. No other interaction effect was significant. Therefore, men with a risk for MD (*n* = 23) showed significantly lower desire for social interaction with the stimulus person (*M* = 12.35, *SD* = 5.75) than men without a risk for MD (*n* = 77; *M* = 15.32, *SD* = 5.77), regardless of the attractiveness of the stimulus person (see Table 3). Moreover, men showed a stronger desire for social interaction with attractive stimulus persons (*M* = 16.88, *SD* = 5.36) than with less attractive ones (*M* = 12.4, *SD* = 5.55).

**Table 3:** Means and standard deviations of participants’ desire for social interaction for all effects

	priming	SP attractiveness	*M*	*SD*	*N*
risk for MD	priming	attractive	21.50	2.12	2
		less attractive	10.22	3.99	9
	no priming	attractive	12.60	6.58	5
		less attractive	12.29	5.96	7
no risk for MD	priming	attractive	18.00	4.56	24
		less attractive	11.06	4.05	17
	no priming	attractive	16.11	5.65	19
		less attractive	14.94	6.72	17

*MD* muscle dysmorphia, *SP* stimulus person

The interaction of the priming of participants with their own body schema and stimulus persons’ attractiveness showed that in the primed group (*n* = 52), the desire for interaction with attractive (*M* = 18.27, *SD* = 4.5) versus less attractive (*M* = 10.77, *SD* = 3.97) stimulus persons was stronger than in the group without the priming (*n* = 48) (attractive stimulus person *M* = 15.38, *SD* = 5.88; less attractive stimulus person: *M* = 14.17, *SD* = 6.5). While the desire for social interaction regarding attractive and less attractive stimulus persons was very similar for the group without the body schema priming, the primed group seemed to have stronger desire for interaction with attractive stimulus persons, on the one hand, and a lower desire for interaction with less attractive persons, on the other hand.

### Moderation analyses

To investigate a potential effect of people’s social comparison tendencies (i.e., to compare upwards or downwards) on their desire for social interaction, three 2 (participants’ risk for MD: high vs. low) × 2 (priming vs. no priming) × 2 (stimulus person attractiveness: high vs. low) analysis of variance on participants’ desire for social interaction with the stimulus person, with one covariate each, were conducted. The tendency for upward social comparisons, for downward social comparisons, and the importance of a positive outcome in social comparisons each functioned as covariates. In a first step, only the main effects were observed.

The tendency for downward comparisons, *F*(1, 99) = 4.43, *p* < .05, η_p_^2^ = .05, and the importance of a positive outcome of social comparisons, *F*(1, 99) = 5.81, *p* < .05, η_p_^2^ = .06, both revealed a significant main effect while the tendency for upward comparisons did not, *F*(1, 99) = .06, *p* = .802, η_p_^2^ = .00. Including each covariate did not change the significant main effects of risk for MD and stimulus persons’ attractiveness. Correlational analysis revealed a significant negative correlation of participants’ desire for social interaction only in case of a tendency for downward comparisons, *r*(98) = −.21; *p* < .05.

In a second step, interaction effects of the three covariates were examined to investigate moderation effects of social comparisons. Neither participants’ upward- or downward comparison tendencies, nor their perceived importance of a (self-rated) positive outcome of social comparisons showed significant interactions with risk for MD, priming, or attractiveness. Therefore, there was no evidence for a moderation effect.

## Discussion

### Muscle dysmorphia

This study set out to investigate the desire for social interaction of men with and without a risk for MD. Additionally, we examined the influence of social comparisons and body schema on the desire for social interaction.

We found that men with a risk for MD (in comparison to men without a risk for MD) showed significantly less desire for social interaction with other persons, regardless of the respective stimulus person’s attractiveness. This is in line with postulations of social withdrawal and isolation in individuals with MD [10]. Moreover, the two groups did not differ in their preferences for social interaction with attractive stimulus persons. According to the “what is beautiful is good” attractiveness stereotype [30], this general preference for contact with attractive candidates is comprehensible in that attractive persons are often perceived as having more socially desirable personality traits and to be leading better lives regarding their partnership and in social and occupational matters. When considering that men’s dating interest is predominantly influenced by physical attractiveness [36], particularly the desire for social contact with attractive opposite-sex stimulus persons might be desired, as this would be more interesting and promising than encounters with less attractive (or same-sex) persons. Since mating motivation and partner choices of men with MD and related body image problems are only scarcely investigated so far, this could be an interesting field for future research.

### Social comparisons

We found no interaction effects regarding men’s tendency for social comparisons. Analyses revealed significant main effects for men’s tendency for downward comparisons and for the importance of a positive outcome of social comparisons, in line with the notion that people who tend to compare downwards try to avoid social threat. That is, the more men generally tended toward downward comparisons, the less interest they showed for social interaction. This is partly in line with Agthe et al. [20], who found that persons engaging in downward comparisons indicated less desire for social interaction with attractive same-sex stimulus persons (who could be a self-threat in social comparison). However, no interactions could be detected in the current study. This might be due to the experimental design of the study. Usually body comparisons are important in the context of MD, body dissatisfaction, and drive for muscularity [14, 24]. In this experiment, only social comparisons regarding occupational success and facial attractiveness have been triggered. Thus, social comparisons on this dimension might not have the same effect as social body comparisons could have had. For future research, the effect of social body comparisons on desire for social interaction in men at risk for MD should be investigated instead of social comparisons regarding occupational success and facial attractiveness.

### Body schema

There was no difference between the priming and non-priming groups regarding men’s desire for social interaction, although negative effects of body confrontation and activation of body schema have been found before [28]. For instance, it was shown that the activation of a negative self-schema in persons with body image problems lead to negative cognitions and emotions [37]. Correspondingly, the exposure to images of idealized male bodies can lead to increased body dissatisfaction in men [38]. All of these aspects are postulated to be related to MD [7], thereby hinting at an enhanced risk of the respective men for social withdrawal or even isolation.

An explanation why we did not find a difference in men who received the body schema priming and men who did not receive it could be that both groups did not differ with regard to body dissatisfaction and thus, negative body schema. This might result from our subclinical sample which might not be as prone to react to body-related priming as a clinical sample. That is, with clinical MD samples, a re-examination of this priming effect on desire for social interaction could be interesting for further investigations, especially when considering that social isolation and regular mirror checking behavior, which might re-activate negative body schema, have been reported before [10].

Interestingly, there was a significant interaction effect found for priming and attractiveness of the stimulus person, leading to the assumption that the activation of ones’ body schema might intensify the desire and non-desire for social interaction with attractive versus less attractive persons. While participants who had not received a priming did not differ, particularly in their desire for social contact with attractive and less attractive persons, those participants who had received the priming reported a much higher desire for social interaction with attractive persons and a much lower desire for social interaction with less attractive persons. Thus, the activation of one’s own body schema apparently intensified the desire to and the avoidance of social interaction with attractive versus less attractive persons. Assuming that the activation of one’s body schema evokes negative feelings, the opposite would have been supposed, a withdrawal from confrontation with attractive persons. On the other hand, negative feelings about ones’ body might motivate upward social comparison processes, represented through the desire for social interaction with attractive stimulus persons. Those upward social comparison processes were postulated to be adopted when self-improvement is intended [20], demonstrating a potentially harmful combination of body dissatisfaction and unfavorable social comparisons, which could lead into a downward spiral of negative body image, social comparison, and potentially harmful behaviors associated with drive for muscularity and MD. These effects could represent a potential maintenance mechanism for body dissatisfaction and require further examination.

### Strengths and limitations

Since most research on MD is using questionnaires and interviews, an experimental study on the effects of MD, social comparison tendencies, and body schema on the desire for social interaction is of great value for this field of research. The results give first insights into potential risks of MD tendencies as well as social comparison processes and body schema on the desire for social interaction and thus, potential maintenance mechanisms of MD and related social withdrawal.

One of the limitations of the current study is its small size. This is because it was very difficult to find a sufficiently large group of persons displaying a risk for MD. Therefore, some results that came relatively close to being statistically significant may not have reached significance, because of the small size of this sub-group. Still, we found some interesting and expected main effects, like the difference between men with and without a risk for MD regarding their desire for social interaction. However, due to the interaction effects, the 2 (group of participant) × 2 (priming condition) × 2 (attractiveness of stimulus person) design with independent measures would have required larger cell sizes. The actual small cell sizes could partly explain some of the insignificant outcomes.

Moreover, precise diagnostic categories for MD in general and accompanying measures in the German language in particular are needed. Although allowing for a first distinction between persons at risk for MD, the screening is still insufficient for comparisons between persons with explicit symptoms of MD and others without these symptoms. Furthermore, another cut-off value could have led to different results, which also hints to the necessity of precise diagnostic categories and adequate measures. Not to forget, a risk for MD does not equal the full picture of MD symptomatology, which might partly explain why we did not find as many differences between both groups as expected. Therefore, the use of translated and validated scales instead of screening instruments could be of use in future research. The same is true for the operationalization of tendencies for upward and downward social comparisons. Instead of using single items, for which no reliability analysis could be conducted, evaluated instruments would be favorable. Thus, there is a need of instruments measuring social comparison tendencies in the German language which could be addressed in future research.

Furthermore, the impact of self-esteem and self-perceived attractiveness would be of interest in future investigations of MD and desire for social interaction. Also sex of stimulus person, according to dating motivation would have been of interest for this study. Since cell sizes were already very small and more variables would have made the analysis even more complex, we decided against their inclusion. For future research, these aspects, especially self-esteem, should be taken into consideration, since MD was repeatedly associated with low self-esteem [39, 40], and self-esteem was related to desire for social interaction [41].

Despite the current limitations, the investigation of MD with regard to social comparisons, body schema and desire for social interaction is worth continuing. Not much research has been done yet in this field, even though social withdrawal and interpersonal problems are severe issues for individuals with body image problems, which have to be recognized, prevented and/or treated, especially considering the relatively high suicide rates of people affected by body dysmorphic disorders or eating disorders like AN [2].

## Conclusion

In conclusion, the current study found differences between men with and without a risk for MD with regard to their desire for social interaction, as well as differences regarding stimulus persons’ attractiveness. Moreover, we found further links of social comparisons with desire for social interaction, and connections of body schema priming and attractiveness of the stimulus person with regard to the desire for social interaction. Future investigations should extend this field of research with clinical populations, for it is highly important for groups that may be small in size, but highly prone to social withdrawal, isolation, and potential pathology, sometimes even including suicidal tendencies.

## Abbreviations

AN: Anorexia nervosa
MD: Muscle dysmorphia
MDI: Muscle Dysmorphia Inventory
MMDI: Muscle Dysmorphic Disorder Inventory
SP: Stimulus person
